# Self-awareness in the retina

**DOI:** 10.7554/eLife.10233

**Published:** 2015-08-25

**Authors:** Andrew M Garrett, Robert W Burgess

**Affiliations:** Jackson Laboratory, Bar Harbor, United States; Jackson Laboratory, Bar Harbor, United Statesrobert.burgess@jax.org

**Keywords:** retina, starburst amacrine cell, synapse elimination, self-recognition, direction selectivity, mouse

## Abstract

Proteins called gamma-protocadherins are essential for the establishment of working circuits of neurons in the retina.

**Related research article** Kostadinov D, Sanes JR. 2015. Protocadherin-dependent dendritic self-avoidance regulates neural connectivity and circuit function. *eLife* **4**:e08964. doi: 10.7554/eLife.08964

**Image** The branches on a starburst amacrine cell avoid other branches from the same cell (cyan), but not those of neighboring cells (pink)

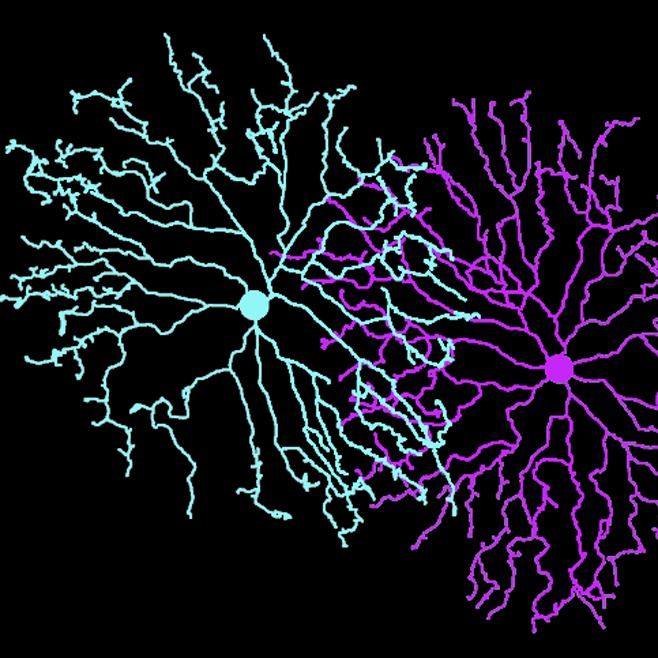

The idea that cell adhesion molecules regulate when and where connections form between different neurons has been in place for over 50 years (Sperry, 1963). However, it has become clear that other cues are important to prevent connections such as synapses forming between the branches of an individual neuron (Zipursky and Sanes, 2010). Now, in *eLife*, Dimitar Kostadinov and Joshua Sanes – both at Harvard University – highlight how this process, which is called self-avoidance, is vital for establishing the neuronal circuitry in the retina of the mammalian eye (Kostadinov and Sanes, 2015).

Self-avoidance can lead to several different arrangements in populations of neurons. For example, cells could be ‘tiled’ so that each cell occupies its own territory (Figure 1A). This presumably requires that the neurons in a given population are ‘repulsed’ by one another, and that the same is true for the branched nerve endings of each individual neuron: this arrangement maximizes the area covered by each cell and minimizes the number of self-crossings.

**Figure 1.**
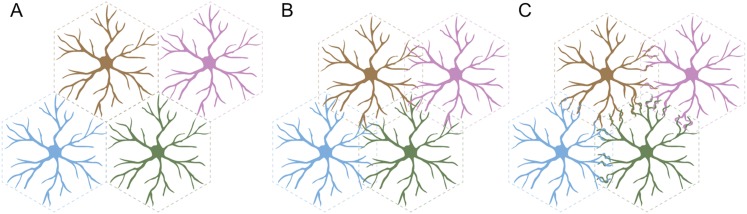
Three arrangements of neurons that require self-avoidance. (**A**) Neurons may occupy discrete, non-overlapping territories; this requires repulsion between cells, and self-avoidance to prevent crossings within the branched nerve endings of a single cell. (**B**) Neurons may avoid their own branches, but be indifferent to those of their neighbors and intermingle without adhering and forming connections. (**C**) Finally, neurons with self-avoidance may nonetheless bind to their neighbors if they also have self/nonself discrimination. Kostadinov and Sanes studied starburst amacrine cells, which display this anatomy with both self-avoidance and self/nonself discrimination mediated by proteins called gamma-protocadherins.

FIGURE CREDIT: GRAPHICS COURTESY OF JESSE HAMMER

Alternatively, the branches of an individual cell could continue to repel each other, but the repulsion between the branches of different cells could be replaced with indifference. This would lead to some overlap between the different cells, but not the formation of connections between them (Figure 1B). In a third arrangement, the branches of a cell could both overlap with and readily bind to the branches of a neighboring cell (Figure 1C). This would require cells to display two phenomena: self-avoidance at the level of individual cells, and the ability to recognize branches belonging to neighboring cells. This is called ‘self/nonself discrimination’. Kostadinov and Sanes studied neurons called starburst amacrine cells (SACs) that display this type of self-avoidance with self/nonself discrimination.

These two phenomena require a diverse code of molecules that distinctly label each cell. Sanes and colleagues had previously demonstrated that proteins called gamma-protocadherins provide this code for SACs (Lefebvre et al., 2012). By combining different versions (or isoforms) of the protein produced from the gamma-protocadherin gene, cells can make thousand of different adhesive labels, and each label only tends to recognize and interact with other labels made from the same isoforms (Schreiner and Weiner, 2010). Molecules called Dscams perform a similar role in *Drosophila* (Zipursky and Grueber, 2013).

Now, Kostadinov and Sanes show the importance of self-avoidance and self/nonself discrimination in the development of neural circuits. First, they either eliminated the expression of all gamma-protocadherins, or allowed the expression of only a single working isoform. In the absence of all gamma-protocadherins, individual SACs began to form synapses with themselves, and formed fewer synapses with neighboring SACs than those in wild type retinas. Furthermore, Kostandinov and Sanes discovered that while many of the synapses that form between SACs are actually eliminated during the normal development of the retina, this loss is reduced if gamma-protocadherins are missing. Finally, and perhaps most significantly, the retinal circuitry failed to work properly because of the anatomical changes caused by the elimination of the gamma-protocadherins.

SACs contribute to a phenomenon called ‘direction selectivity’ that involves neurons in the retina only firing when they detect an object moving in a specific direction. The degree of direction selectivity in the cells that receive signals from the SACs was severely reduced when the SACs lacked gamma-protocadherins.

Direction selectivity was also reduced when SACs expressed a single gamma-protocadherin isoform. However, in this case, individual cells still exhibited self-avoidance and formed branched nerve endings. However, self/nonself discrimination was perturbed, which adversely affected the connections with neighboring SACs. As such, the loss of all gamma-protocadherins had a slightly different effect compared to the loss of all but one gamma-protocadherin. This likely reflects the fact that SACs with a single gamma-protocadherin look more like wild type cells than cells with none.

Kostandinov and Sanes also found that either eliminating all gamma-protocadherins, or all but one, did not affect the connections between the SACs and other cell types (which either send signals to, or receive signals from, the SACs). Therefore, the differences in direction selectivity reflect problems within the SAC network itself. Thus, these findings offer a clear and comprehensive characterization of the consequences of losing self-avoidance and self/nonself discrimination, in terms of both how the SACs connect with other cells, and how this affects the way in which they operate.

While the importance of self-avoidance and self/nonself discrimination in SACs is clear, many questions remain. First, how many cell types use gamma-protocadherins in this way? It has been suggested that cerebellar Purkinje cells might do so too (Lefebvre et al., 2012), but both SACs and Purkinje cells are rather specialized cells and may not represent a typical neuron. Furthermore, several other proteins can act as cues to promote self-avoidance and control the shaping of neural circuits (Fuerst et al., 2008; Matsuoka et al., 2012); could these molecules also be involved and could the same molecules have different roles in specific cell types? Finally, how do ‘adhesion molecules’ recognize each other in a way that, depending on the context, sometimes results in avoidance and not adhesion (Yamagata and Sanes, 2008; Schreiner and Weiner, 2010)? These and other questions have now become all the more important for understanding how the formation of neural circuits is optimized.
